# Clinical pharmacist interventions in medication review for medication optimization in older hospitalized adults with mental disorders and somatic comorbidities: evidence from retrospective study

**DOI:** 10.3389/fphar.2025.1667584

**Published:** 2025-09-01

**Authors:** Matej Stuhec, Nusa Svetanic, Tomaz Vovk, Anteja Gorjan Gazdag, Zala Cuk, Borjanka Batinic

**Affiliations:** ^1^ Department of Pharmacology & Department of Clinical Pharmacy, Medical Faculty Maribor, University of Maribor, Maribor, Slovenia; ^2^ Department of Clinical Pharmacy, Ormoz’s Psychiatric Hospital, Ormoz, Slovenia; ^3^ Department of Biopharmaceutics and Pharmacokinetics, Faculty of Pharmacy, University of Ljubljana, Ljubljana, Slovenia; ^4^ Clinic of Psychiatry, University Clinical Centre of Serbia, Belgrade, Serbia; ^5^ Department of Psychology, Faculty of Philosophy, University of Belgrade, Belgrade, Serbia

**Keywords:** psychiatry, clinical pharmacy, hospital, medication review, drug-related problem

## Abstract

**Introduction:**

Collaboration with clinical pharmacists in the medication review process can potentially optimize pharmacotherapy for elderly patients with mental disorders and somatic comorbidities.

**Aim:**

This study aimed to evaluate the impact of clinical pharmacists’ recommendations during medication reviews, including changes in the number of medications, potentially inappropriate medications (PIMs), potential drug-drug interactions (DDIs), and adherence to treatment guidelines.

**Methods:**

A retrospective, non-interventional study was conducted in a psychiatric hospital in Slovenia. The study included inpatients aged ≥65 years with mental disorders who were referred for medication reviews between 2013 and 2018 and had at least one therapy modification related to somatic comorbidities (heart failure, arterial hypertension, or diabetes). Clinical pharmacists conducted type 3 medication reviews (advanced medication reviews), as defined by the Pharmaceutical Care Network Europe They recorded their recommendations in the hospital’s electronic system immediately after completing the medication review. Data from before (before the medication review) and after (outcomes extracted from the electronic system at discharge) were systematically reviewed. The primary outcomes were changes in the number of medications, PIMs, and DDIs before and after the intervention. The secondary outcome was adherence to treatment guidelines for somatic comorbidities (heart failure, arterial hypertension, and diabetes).

**Results:**

The study included 100 inpatients with a mean age of 78.1 years (SD = 6.78). The total number of medications decreased by 6.6% (from 1,144 to 1,068; p < 0.001), with an acceptance rate of 59.2%. After the review, X-type DDIs decreased by 75.8% (from 33 to 8; p < 0.001), and D-type DDIs decreased by 56.9% (from 188 to 81; p < 0.001). The number of PIMs also significantly decreased (p < 0.001), with reductions of 29.5% (from 308 to 217) based on the Priscus List and 17.5% (from 343 to 283) according to the Beers Criteria. Adherence to treatment guidelines for somatic comorbidities improved significantly (from 3.3%–13.2% to 50.0%–72.6%; p < 0.001).

**Conclusion:**

This study demonstrates that interventions by clinical pharmacists during the medication review process effectively reduced the number of medications, PIMs, and DDIs while significantly improving adherence to treatment guidelines.

## 1 Introduction

Most inpatients in psychiatric hospitals have multiple comorbidities, both somatic and psychiatric (Goldman et al., 2020). Somatic comorbidities are a leading cause of death in these patients and significantly increase the risk of rehospitalisation. This correlation has been extensively studied using real-world data from patients with schizophrenia, where cardiovascular and cerebrovascular diseases were identified as the primary causes of death (Taipale et al., 2020; Bitter et al., 2017).

Despite these findings, monitoring and screening for somatic conditions in these patients remain inadequate, with only a minority of comorbid cases being appropriately screened and treated (Soerensen et al., 2016). Furthermore, psychiatric inpatients face numerous drug-related problems (DRPs), including drug-drug interactions (DDIs), potentially inappropriate medications (PIMs), polypharmacy, and low adherence to treatment guidelines (Soerensen et al., 2016). Research has revealed that 59.0% of adult inpatients with mental disorders are prescribed PIMs. This includes excessively high doses (16.0%), DDIs (36.0%), and significant polypharmacy (Soerensen et al., 2016). These findings underscore the need for interdisciplinary treatment strategies to enhance pharmacotherapy for this population. Effective management of somatic comorbidities is a critical component of pharmacotherapy optimization (Farley et al., 2012; Stuhec et al., 2024). Unfortunately, many of these patients are not adequately screened or treated (Stuhec et al., 2024). Moreover, these scenarios are often excluded from randomized controlled trials, guidelines, and meta-analyses (Keepers et al., 2020), further highlighting the necessity of interdisciplinary approaches involving various healthcare specialists.

Clinical pharmacists are well-trained healthcare professionals with a focus on pharmacotherapy management and improving clinical outcomes (Stuhec et al., 2024). Medication review represents one of the key clinical pharmacy services, which has been developed and recognised at the international level (Keepers et al., 2020). Clinical pharmacists provide advanced medication review after direct conversation with patients, lab tests, and datasets checking, including medication history (Robberechts et al., 2024). Clinical pharmacists could also monitor patients, which is highly recommended and therefore provide continuation of care. This approach has been widely studied in some countries, including the United Kingdom and Slovenia (Stuhec, 2021; Stuhec, 2025). In a Slovenian retrospective study, including patients with mental disorders in primary care, Stuhec and Zorjan showed positive effects on DRPs (246 patients) (Stuhec and Zorjan, 2022). The clinical pharmacists proposed 374 interventions in psychopharmacotherapy. The general practitioners accepted 45.2% of them. Accepting clinical pharmacist recommendations reduced the total number of medications by 7.5% from 13.4 to 12.4 per patient (p < 0.05), the total number of prescribed PIMs by 21.8% from 312 to 244 (p < 0.05), the number of potential DDIs by 54.9% from 71 to 31 (p < 0.05) and also improved treatment guidelines adherence for antidepressants and antipsychotics (p < 0.05). The following study by Stuhec et al. in the Slovenian primary care settings included 48 patients with mental disorders (79.4 years, SD = 8.13) receiving a total of 558 medications (155 for the treatment of mental disorders) (Stuhec and Lah, 2021). The number of medications decreased by 9.5% following the clinical pharmacist’s medication review. The clinical pharmacist proposed 198 interventions related to psychotropics, of which 108 (54.5%) were accepted by the general practitioners. All accepted (99.1%) interventions, except one, were maintained 6 months after the proposed interventions. They led to a significant decrease in the total number of medications, PIMs, and potential DDIs and improved adherence to treatment guidelines (Stuhec and Lah, 2021). Although these results are important, the authors did not include psychiatric hospital settings and inpatients with somatic comorbidities, and the consequences in psychiatric hospitals remain unknown.

Although this approach is widely used in somatic hospitals and ambulatory services, it is not as widely adopted in psychiatric hospitals across Europe (Stuhec et al., 2023; Urbańczyk et al., 2023). Clinical pharmacists are not well-integrated, and their services remain unstandardized and unreimbursed in most countries (Stuhec et al., 2023). In a previous study, we explored the impact of clinical pharmacists’ involvement in daily ward rounds at a psychiatric hospital (excluding medication reviews) for patients with somatic comorbidities and observed positive results (Stuhec et al., 2024). Clinical pharmacists participated in an interdisciplinary team, making 280 recommendations related to drug-related problems (DRPs), averaging 1.5 recommendations per patient. Among these, 154 (55.0%) were categorized as expressed DRPs, while 127 (45.0%) were identified as potential DRPs. Following pharmacists’ recommendations, 133 (86.4%) expressed DRPs were resolved successfully. The initial acceptance rate of recommendations was 88.9% (N = 249) at discharge, but decreased to 63.2% (N = 177) 3 months post-discharge. For somatic conditions specifically, the acceptance rate at discharge was 87.8% (N = 122), dropping to 59.0% (N = 82) 3 months later (Stuhec et al., 2024). Adherence to treatment guidelines for somatic comorbidities, including hypertension, heart failure, diabetes, and pain management, improved significantly. Despite these positive outcomes, a key limitation was that clinical pharmacists provided single-point interventions rather than comprehensive medication reviews.

Collaborating with clinical pharmacists in the medication review (PCNE type 3) process is a promising approach to optimizing pharmacotherapy in these patients. However, this approach has not been widely studied in psychiatric inpatients with somatic comorbidities (Stuhec et al., 2024). This study aims to evaluate the impact of clinical pharmacists’ recommendations during medication reviews on pharmacotherapy optimization in this population. We hypothesize that these recommendations will positively influence predefined outcomes, including the number of medications, PIMs, potential DDIs, and adherence to treatment guidelines.

## 2 Materials and methods

### 2.1 Setting

This study was conducted at the Ormož Psychiatric Hospital, a 100-bed facility in Slovenia that provides outpatient and inpatient services. Clinical pharmacists have been integral members of the interdisciplinary ward team since 2010. They offer clinical pharmacy services, including medication reviews, daily ward interventions, and specialized support across all hospital wards (e.g., psychogeriatric, closed and open wards, and the ward for addiction treatment).

As part of the interdisciplinary team, clinical pharmacists perform daily interventions on the ward—a role previously studied and published by our research team in another paper (Stuhec and Zorjan, 2022). Comorbidities in inpatients are assessed solely by ward psychiatrists and internal medicine doctors, if required. More complex cases are transferred to a general hospital for somatic care. In this context, clinical pharmacists are crucial in managing comorbidities by conducting daily medication reviews and providing other essential interventions. Clinical pharmacy services in Slovenia operate under the hospital pharmacy, which coordinates and oversees these activities (Urbańczyk et al., 2023).

### 2.2 Type of intervention

Medication reviews have been a part of Slovenian hospital practice for over 20 years, with clinical pharmacists regularly providing this service. They conduct type 3 medication reviews (advanced medication reviews) as defined by the Pharmaceutical Care Network Europe (PCNE). This service has been standardized in Slovenia, with a defined content and duration of 60 min (Stuhec, 2021; Urbańczyk et al., 2023; PCNE, 2013). Advanced medication reviews are based on a patient’s medication history, relevant patient information, and clinical data. They address all critical aspects outlined by the PCNE, including drug interactions, side effects, unusual dosages, adherence issues, drug-food interactions, effectiveness concerns, over-the-counter medication problems, unindicated medications, missing indications, and dosage issues (Stuhec, 2021; Urbańczyk et al., 2023; PCNE, 2013). The Slovenian Pharmaceutical Chamber adopted a standard operating procedure for medication reviews, which was included in the Slovenian Pharmacy Act 2017. Only clinical pharmacist specialists with 3 years of specialized training are authorized to provide this service. During a medication review, clinical pharmacists recommend changes to therapy based on patient consultations and current pharmacotherapy (Stuhec, 2021; Stuhec, 2025; Urbańczyk et al., 2023). These recommendations are then discussed with physicians, who make the final decisions, as clinical pharmacists do not have prescribing rights.

Throughout the study, clinical pharmacists conducted medication reviews in a consistent format without any procedural changes. Medication reviews were documented in the hospital’s electronic system and included in discharge letters. This service is exclusively provided for inpatients on behalf of psychiatrists. Unlike in primary care settings, hospital-based medication reviews have not been reimbursed (Urbańczyk et al., 2023).

Clinical pharmacists recorded their recommendations in the hospital’s electronic system immediately after conducting the medication review. Data from the before (before the medication review) and after (outcomes extracted from the electronic system at discharge) were systematically reviewed, ensuring consistency and high-quality data for all recommendations.

### 2.3 Inclusion and exclusion criteria

The study included patients hospitalized at the Ormož Psychiatric Hospital who underwent a medication review by a clinical pharmacist between 1 January 2013, and 31 December 2018. The population consisted of older adults (aged 65 years or older) with polypharmacy (five or more medications). Patients should had at least one mental disorder as defined by the 10th revision of the International Statistical Classification of Diseases and Related Health Problems (ICD-10) (WHO, 2025). The inclusion criterion was that the clinical pharmacist had made at least one suggestion for modifying the therapy of somatic comorbidities, such as arterial hypertension, heart failure, or diabetes, during the medication review. The pharmacist’s suggestions regarding these conditions were later evaluated for adherence to treatment guidelines. If multiple medication reviews were conducted for a single patient during the study period, only the first review was included, ensuring the number of medication reviews matched the number of included patients. Only three main types of interventions were analyzed in the medication reviews: discontinuation of medications, initiation of new medications, and dose adjustments. Reviews and interventions provided by clinical pharmacists outside the scope of this analysis were excluded.

The study included only three somatic comorbidities: arterial hypertension, heart failure, and diabetes. These were selected based on their prevalence in the hospital population, as identified through hospital database records and a review of pharmacotherapy evaluations conducted over the past 10 years. Neurological comorbidities were excluded from the study, as they were managed separately by a neurologist when necessary. For this reason, we chose to focus on these conditions in our paper. At the time of the study, comorbidities were assessed only by hospital psychiatrists and clinical pharmacists. If requested by a psychiatrist, pharmacist recommendations were reviewed by an internal medicine consultant. In cases of more complex issues, patients may be referred to a somatic hospital.

### 2.4 Outcomes

The primary outcomes of the study were changes in the number of medications, PIMs, and DDIs before and after the intervention. DDIs were identified using the Lexicomp Online^®^ database, categorized as X-type (contraindicated) and D-type (major). To identify PIMs in elderly patients, we referred to the latest Priscus List 2.0 and the updated Beers Criteria, published in 2023 (Mann et al., 2023; By the 2023 American Geriatrics Society Beers Criteria^®^ Update Expert Panel, 2023). Medications prescribed on an as-needed basis were excluded from the analysis due to a lack of information on their frequency or daily dosage. However, if a medication was considered a PIM regardless of its dosage, it was included even if prescribed as needed.

We reviewed the prescription histories of PIMs and found that all patients were treated for durations exceeding those specified in the PIM lists, which led to their classification as PIMs. Conversely, if a medication was newly introduced during the medication review and classified as a PIM only with long-term use, it was not evaluated as part of the review. When applying the Beers Criteria, only medications listed in Table 2 of the 2023 update were considered (By the 2023 American Geriatrics Society Beers Criteria^®^ Update Expert Panel, 2023).

The secondary outcome was adherence to treatment guidelines for somatic comorbidities. Adherence was evaluated based on European treatment guidelines for heart failure, hypertension, and diabetes: ESC Guidelines for the diagnosis and treatment of acute and chronic heart failure, ESC Guidelines for the management of elevated blood pressure and hypertension and Management of Hyperglycemia in Type 2 Diabetes by the American Diabetes Association (ADA) and the European Association for the Study of Diabetes (EASD) (McDonagh et al., 2021; Davies et al., 2022; McCarthy et al., 2025). Pharmaceutical suggestions for each diagnosis were collected from medication reviews and assessed to determine their alignment with the guidelines. We documented identified discrepancies, the pharmacist’s suggestions, and whether the physician implemented those suggestions (absolute difference). For heart failure, we did not have specific information on the subtype of the disease. Therefore, we assumed all patients had heart failure with reduced ejection fraction (HFrEF). In cases where patients with heart failure also had a diagnosis of arterial hypertension, we additionally evaluated adherence to hypertension guidelines. This approach was taken because these conditions are closely related, and the therapeutic drug classes often overlap. Adherence to treatment guidelines for heart failure, hypertension, and diabetes was illustrated case by case in a table format.

### 2.5 Data collection

Data were collected from hospital medication reviews. Initially, we gathered data describing the sample before the clinical pharmacist’s intervention. For this purpose, we used information from the medication reviews conducted before the clinical pharmacist’s (before). Subsequently, we reviewed the medication reviews and discharge summaries to calculate differences (after). To ensure anonymity, we encrypted the data for patients, clinical pharmacists, and physicians.

The data were organized into a Microsoft Excel 2016 worksheet. We also examined the number and types of therapy change suggestions made by the clinical pharmacist. If a change in medication was suggested, it was considered both a discontinuation of the existing medication and an introduction of a new one. Changes to the treatment regimen included adjustments in dosage (increasing or decreasing) and dosing frequency (e.g., from once to twice daily).

Data collection was performed retrospectively by reviewing the hospital’s electronic medication records after ethical approval. Three researchers (N.S., Z.C., and A.G.G.), who were not clinical pharmacists and were uninvolved in daily rounds, collected and extracted the data in 2024 after obtaining ethical approval. Ethical approval was obtained from the National Medical Ethics Committee in Slovenia on 6 March 2024 (approval number: 0120-544/2023-2711-6). Other researchers contributed to various aspects of the study, including data interpretation (B.B., T.V., and M.S.). The research team included pharmacists and a medical specialist (a psychiatrist, B.B.), who verified the data quality during collection and provided external review to minimise bias.

### 2.6 Statistics

Descriptive statistics were used to summarize the characteristics of the selected population. To describe the sample numerically, we reported sums, medians, means, and minimum and maximum values. Before further analysis, we tested the normality of our variables using the Shapiro-Wilk normality test. For statistical analysis, paired samples t-tests and Wilcoxon signed-rank tests were applied. McNemar’s test for related samples assessed adherence to treatment guidelines. We also examined correlations between certain variables using Spearman’s correlation test. A significance level of p < 0.05 was used for all statistical tests. Patients with any missing data were excluded from the study. The sample included all possible medication reviews during the study period. The sample size was determined based on previous studies conducted in primary care settings (Stuhec and Lah, 2021; Stuhec et al., 2019), as well as a power analysis conducted using the G*Power^®^ software. The calculation was based on the following parameters: alpha = 0.05, power (1–β) = 0.90, and an effect size of 0.3, resulting in a required total sample size of 97. The Bonferroni correction was applied to account for the multiple comparisons problem. Given that seven different comparisons were performed, the significance threshold was adjusted to p = 0.05/7 = 0.0071. Effect sizes were calculated for continuous variables using Cohen’s d, and for categorical variables using odds ratios (ORs), both with 95% confidence intervals. The study adhered to the STROBE (Strengthening the Reporting of Observational Studies in Epidemiology) guidelines (von Elm et al., 2008). Data analysis was performed using Microsoft Office Excel^®^ 2016 and IBM SPSS Statistics version 26.

## 3 Results

### 3.1 Baseline characteristics

A clinical pharmacist conducted 106 medication reviews during the study period, of which 100 (94%) were eligible for inclusion because the patients also had somatic comorbidities. A total of 100 patients were included in the study. Women represented 73.0% (N = 73) of the participants, and men represented 27.0% (N = 27). The mean age of the patients was 78.1 years (SD = 6.8).

**FIGURE 1:**
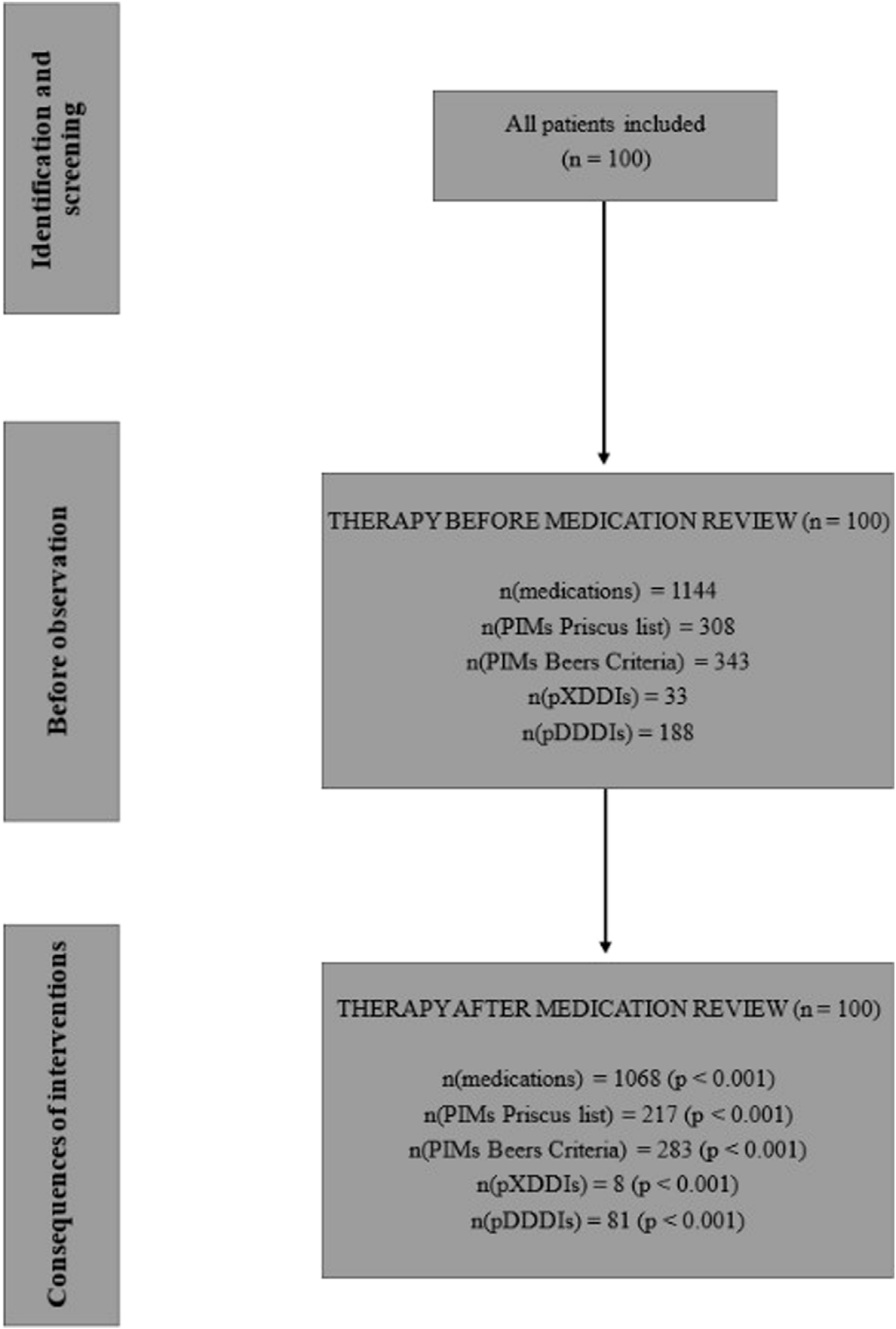
Flowchart.

In total, the clinical pharmacist made 559 recommendations. On average, the pharmacist made 5.6 recommendations for therapy changes per patient (median = 5), with a maximum of 13 recommendations for a single patient. The most common recommendation was drug discontinuation (49.0%; N = 274), followed by drug initiation (24.5%; N = 137) and dose or frequency adjustments (26.5%; N = 148). Out of 559 recommendations, 331 were accepted by the psychiatrists, indicating an acceptance rate of 59.2%. The results of the impact of pharmacist interventions are presented in Figure 1.

### 3.2 Primary outcomes (drug-related problems)

#### 3.2.1 General results

Altogether, the patients were prescribed 1,144 medications (an average of 11.4 medications per patient, median = 11), with a maximum of 18 medications prescribed to one patient. The majority of patients received between 8 and 11 drugs (N = 45; 45.0%). After the medication reviews, the total number of medications decreased by 76, representing a 6.6% reduction (p < 0.001). Patients received a total of 1,068 medications, with an average of 10.7 medications per patient (median = 11). The number of patients receiving more than 11 medications decreased, while the number of patients with fewer prescribed medications increased. The calculated effect size (Cohen’s d) was −0.27, 95% CI (−0.667; −0.121).

#### 3.2.2 Potentially inappropriate medications in the elderly

**FIGURE 2:**
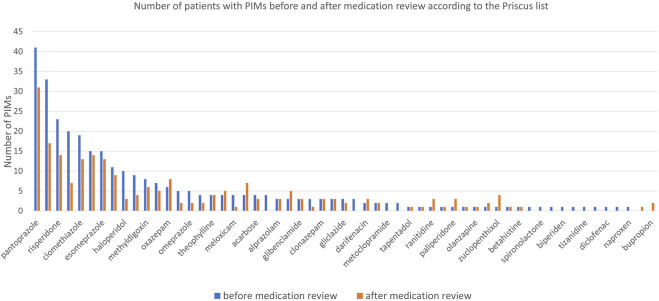
Number of patients with PIMs before and after medication review for analysed medications according to the Priscus list.

**FIGURE 3:**
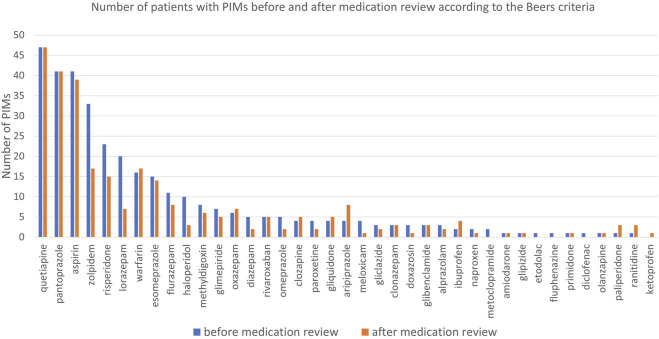
Number of patients with PIMs before and after medication review for analysed medications according to the Beers Criteria.

Before the medication review, patients received on average 3.1 PIMs based on the Priscus list and 3.4 based on the Beers Criteria. Most PIMs, according to both lists, were psychotropics. After the pharmacist’s interventions, the number of PIMs according to the Priscus list decreased by 29.5% (from 308 to 217; p < 0.001) and by 17.5% according to the Beers Criteria (from 343 to 283; p < 0.001). The reduction in PIMs is shown in Figure 2 (Priscus list) and Figure 3 (Beers Criteria). The calculated effect sizes (Cohen’s d) were −0.53 (95% CI: 0.928; −0.131) for the Beers Criteria and −0.67 (95% CI: 1.073; −0.267) for the PRISCUS list. The PIMs identification process has been thoroughly documented in the Supplementary Material.

#### 3.2.3 Potential drug-drug interactions

Regarding the X-type DDIs detected, the pharmacist made 27 recommendations, of which 22 (81.5%) were accepted by the psychiatrists. Potential X-type DDIs decreased from 33 to 8 after the medication review, representing a 75.8% reduction (p < 0.001). The drug most frequently involved in X interactions was quetiapine (42.4% of all X-type DDIs), interacting with eight different medications. The clinical pharmacist also contributed to a reduction of potential D-type DDIs, which decreased from 188 before the medication review to 81 after (a 56.9% reduction; p < 0.001). Before the medication review, 69.0% of patients (N = 69) had at least one D-type DDI, and after the pharmacist interventions, this number decreased to 50.0% (N = 50). The calculated effect sizes (Cohen’s d) were −0.464 (95% CI: 0.861; −0.067) for X-type DDIs and −0.676 (95% CI: 1.079; −0.273) for D-type DDIs.

### 3.3 Secondary outcomes

Among the patients, 34 had a diagnosis of heart failure. The clinical pharmacist made 71 suggestions for these patients, of which 64.8% (n = 46) were accepted. As a result, guideline adherence in heart failure therapy improved from 9.9% to 71.8% (p < 0.001)—Tables 1–3 present non-compliance with the guidelines and proposed changes. The effect size (OR) was 23.3 (95% CI: 9.1; 59.5) for heart failure.

**TABLE 1:** Review of heart failure treatment, including clinical recommendations.

Case number	Problem	Clinical pharmacist’s recommendations	Acceptance
1, 2, 3, 4	Not receiving a β-blocker	β-blocker initiation	NO, YES, NO, YES
5	Nonselective β-blocker	Switching to bisoprolol	YES, YES
6	Not receiving spironolactone	Spironolactone initiation	NO
7	Not receiving an ACE inhibitor	ACE inhibitor initiation	NO
8	Inappropriate dosing of perindopril	Dose adjustment	YES
9, 10, 11, 12, 13, 14, 15, 16, 17, 18, 19	Inappropriate dosing of β-blocker	Increase β-blocker dose	YES, YES, YES, YES, NO, NO, NO, YES, YES, YES, YES
20	Use of furosemide (no edema)	Furosemide discontinuation	NO
21	Use of furosemide (no edema)	Reduce furosemide dose	YES
22	Use of torasemide (no edema)	Reduce torasemide dose	YES
23	Use of furosemide + risperidone (D interaction)	Reduce furosemide dose	NO
24	Use of furosemide + risperidone (D interaction)	Furosemide discontinuation	NO
25	Valsartan once daily	Valsartan twice daily	YES
26, 27	Bisoprolol twice daily	Bisoprolol once daily	NO, YES
28	hypotension	Discontinuation of medication	YES
29, 30, 31, 32, 33	Hypotension	Reduce dose of medication	NO, NO, YES, YES, YES
34, 35	Hypertension	Increase dose of medication	YES, YES
36, 37, 38	Use of ACE inhibitor + sartan combination	Discontinuation of medication	YES, YES, NO
39	Use of methyldigoxin	Switching to bisoprolol	YES, YES
40	Use of methyldigoxin	Methyldigoxin discontinuation	NO
41, 42, 43, 44, 45	Use of less appropriate ACE inhibitor in renal failure	Switching to a more suitable ACE inhibitor	YES, YES, YES, YES, YES, YES, YES, NO, YES, YES
46	Ramipril 15 mg/day in renal failure	Reduce ramipril dose	NO
47, 48	Use of lercanidipine in renal failure	Switching to lacidipine/amlodipine	NO, NO, YES, YES
49, 50	Inappropriate dosing of furosemide in renal failure	Increase furosemide dose	YES, YES
51	Inappropriate dosing of torasemide in renal failure	Increase torasemide dose	YES
52, 53, 54	Use of spironolactone in renal failure	Spironolactone discontinuation	YES, YES, YES
55	Missed medication	Torasemide initiation	YES

Regarding arterial hypertension, 106 recommendations were proposed in 65 patients, and the psychiatrists accepted 66.0% (n = 70). Adherence to hypertension treatment guidelines improved from 13.2% to 72.6% (a 59.4% increase; p < 0.001). The effect size (OR) for arterial hypertension was 17.5 (95% CI: 8.6; 35.4).

**TABLE 2:** Review of arterial hypertension treatment, including clinical recommendations.

Case number	Problem	Clinical pharmacist’s recommendations	Acceptance
1, 2, 3, 4, 5	High blood pressure	Increase dose of medication	YES, NO, YES, YES, NO
6, 7, 8, 9, 10	High heart rate and blood pressure	Increase β-blocker dose	NO, YES, YES, YES, YES
11, 12	High heart rate and blood pressure	β-blocker initiation	YES, YES
13, 14, 15, 16, 17, 18, 19, 20, 21	Low blood pressure	Discontinuation of medication	NO, YES, YES, NO, YES, YES, YES, NO, YES
22, 23, 24, 25	Low blood pressure	Reduce dose of medication	NO, YES, NO, YES
26	Amlodipine twice daily	Amlodipine once daily	YES
27, 28, 29	Bisoprolol twice daily	Bisoprolol once daily	NO, NO, YES
30	Use of metoprolol	Switching to ramipril	NO, NO
31, 32	Use of bisoprolol	Switching to ramipril	NO, NO, NO, NO
33, 34	Use of bisoprolol	Bisoprolol discontinuation	YES, YES
35, 36	Use of non-selective β-blocker (carvedilol)	Switching to bisoprolol	YES, YES, YES, NO
37	Use of spironolactone	Switching to ramipril	NO, NO
38	Use of doxazosin	Switching to amlodipine	YES, YES
39	Use of ACE inhibitor + sartan combination	Switching to amlodipine	YES, YES
40, 41, 42, 43, 44, 45, 46, 47	Use of less appropriate ACE inhibitor in renal failure	Discontinuation and initiation of more appropriate one	YES, NO, YES, YES, YES, NO, YES, YES, YES, YES, YES, YES, YES, YES, YES, YES
48, 49	Inappropriate dosing of perindopril in renal failure	Reduce perindopril dose	NO, YES
50	Use of fixed combination of 10 mg ramipril and 5 mg amlodipine in renal failure	Switching to combination with 5 mg ramipril and 10 mg amlodipine	YES, YES
51, 52	Use of hydrochlorothiazide in renal failure	Hydrochlorothiazide discontinuation	YES, YES
53	Use of fixed combination of losartan and hydrochlorothiazide in renal failure	Discontinuation and losartan initiation	YES, YES
54, 55, 56	Use of spironolactone in renal failure	Spironolactone discontinuation	YES, YES, YES
57	Taking too low dose of furosemide (due to renal failure)	Increase furosemide dosage	YES
58, 59	Inappropriate dosing of torasemide in renal failure	Increase torasemide dose	YES, NO
60	Use of losartan in liver failure	Losartan discontinuation	NO
61	Use of perindopril in hyperkalemia	Switching to amlodipine	YES, YES
62	Use of furosemide + risperidone (D interaction)	Reduce furosemide dose	NO
63	Use of furosemide + risperidone (D interaction)	Furosemide discontinuation	NO
64, 65, 66, 67, 68, 69, 70, 71, 72	Use of furosemide (no edema, ineffective dose)	Furosemide discontinuation	NO, YES, NO, NO, YES, YES, YES, YES, NO
73	Use of furosemide (no edema)	Reduce furosemide dose – furosemide as needed only	YES

For patients with a diagnosis of diabetes (n = 44), the pharmacist was followed by psychiatrists in 46.7% (14 out of 30 suggestions), which contributed to an improvement in adherence to diabetes treatment guidelines from 3.3% to 50.0% (a 46.7% increase; p < 0.001). The effect size (OR) was 29.0 (95% CI: 3.5; 241.1) for diabetes.

**TABLE 3:** Review of diabetes treatment, including clinical recommendations.

Case number	Problem	Clinical pharmacist’s recommendations	Acceptance
1,2	Low blood sugar	Reduce insulin dose	YES, YES
3	Low blood sugar	Gliclazide discontinuation	YES
4, 5, 6, 7, 8, 9, 10	High blood sugar	Increase insulin dose	NO, NO, NO, YES, YES, YES, YES
11	High blood sugar	Increase metformin dose	NO
12	Unregulated blood sugar	Redistribution of insulin	NO
13	Mmetformin use in renal failure	Metformin discontinuation	YES
14, 15	Mmetformin use in renal failure	Switching to gliquidone	YES, YES, NO, NO
16	Metformin use in renal failure	Switching to vildagliptin	NO, NO
17	Glimepiride use in renal failure	Switching to gliquidone	YES, YES
18	Use of metformin and acarbose	Increase metformin dose and acarbose discontinuation	NO, NO
19	Use of insulin and acarbose	Increase insulin dose and acarbose discontinuation	YES, YES
20	Use of acarbose	Switching to metformin	NO, NO
21	Underuse of metformin, risk of hypoglycemia	Increase metformin dose and gliclazide discontinuation	NO, NO

## 4 Discussion

This is the first before/after study to examine the role of clinical pharmacists in conducting medication reviews for this population in a psychiatric hospital. The analysis highlights several significant findings that could enhance the involvement of clinical pharmacists in psychiatric hospitals and support the introduction of new reimbursed pharmaceutical services. Our results confirm the hypothesis that clinical pharmacists’ interventions positively impact reducing medication-related problems and improving adherence to treatment guidelines.

The study demonstrates a positive impact on the number of medications prescribed, with a 6.6% reduction, with an acceptance rate of 59.2% for pharmacist recommendations. Polypharmacy is particularly prevalent in this population, and collaboration with clinical pharmacists through medication reviews led to a reduction in the number of medications, which was observed in the primary care (Stuhec et al., 2023; Stassen et al., 2022). However, this acceptance rate is lower than the 88.9% observed during daily interdisciplinary ward rounds in a psychiatric hospital, likely because medication reviews are broader and more complex than single interventions. Polypharmacy is associated with poorer clinical outcomes, as evidenced by systematic reviews showing links to increased hospitalizations and inappropriate prescribing (Davies et al., 2020). Our findings align with trends observed in Slovenian primary care settings, where hospital-based multidisciplinary collaboration appears to improve acceptance rates compared to primary care settings, where general practitioners often work in isolation (Stuhec and Zorjan, 2022; Stuhec and Lah, 2021).

Clinical pharmacists’ interventions significantly reduced PIMs and DDIs. For instance, the number of PIMs, such as benzodiazepines (including zolpidem) and proton pump inhibitors (PPIs) like pantoprazole, decreased notably. Long-term PPI use, identified as a PIM in updated Priscus criteria, was reduced by 24.4%. These results compare favourably with reductions reported in similar studies, such as a 2020 Slovenian study with a 21.1% PIM reduction using the Priscus list (Stuhec and Nemec, 2023). Additionally, DDIs were minimized, contributing to safer pharmacotherapy, as shown in previous Slovenian studies (Stuhec and Zorjan, 2022; Stuhec and Lah, 2021; Stuhec and Nemec, 2023). In one notable case, a significant DDI involving quetiapine and trazodone was resolved, demonstrating the pharmacist’s critical role despite deviations from specific recommendations. In our study, the reduction according to the Priscus list was slightly higher (29.5%), while the reduction according to the Beers Criteria was comparable (17.5%). These results are also in line with a huge retrospective study done in Switzerland, including 1,726,491 patients in primary care, where PPIs consumption is increased over the years (incidence of PPIs increased from 4.8% (2013) to 6.4% (2017), (p = <0.001)) (Muheim et al., 2021). The authors provided evidence that one of the most prescribed drug groups is commonly prescribed inappropriately in the general population and that this trend is increasing (Muheim et al., 2021). These findings collectively reinforce the importance of medication review as a tool for reducing risks and enhancing the safety of pharmacotherapy, which aligns with previous results (Stuhec, 2021).

The last important finding relates to improved treatment adherence for somatic comorbidities. Somatic comorbidities are the leading cause of death in many mental disorders, particularly schizophrenia (Taipale et al., 2020; Bitter et al., 2017). In this context, collaboration with clinical pharmacists can lead to better outcomes and assist psychiatrists in managing such complex cases. Adherence to treatment guidelines for heart failure increased significantly, from 9.9% to 71.8%. Clinical pharmacists made numerous recommendations for initiating and titrating β-blockers. These medications should be titrated to the maximum tolerable doses, a practice often lacking in real-world clinical settings, despite evidence that higher doses reduce mortality in these patients (McDonagh et al., 2021; Ajam et al., 2018). Additionally, clinical pharmacists recommended initiating spironolactone in some instances, which also decreases mortality in heart failure patients (McDonagh et al., 2021). Other recommendations included initiating, switching, and/or titrating ACE inhibitors, often based on considerations of kidney function. Nearly all recommendations were aligned with treatment guidelines (McDonagh et al., 2021). These findings are consistent with previous studies in Slovenian primary care, where Stuhec and Gorenc described the impact of clinical pharmacists on adherence to heart failure treatment guidelines (Stuhec et al., 2019). We also observed several recommendations for the prudent use of diuretics, focusing on dose adjustments in cases of kidney failure and discontinuation as a long-term treatment. This, too, reflects adherence to established recommendations and guidelines (McDonagh et al., 2021).

For arterial hypertension, the main issues included both uncontrolled high and low blood pressure. In such cases, dose increases or reductions of antihypertensive drugs were recommended. A common problem was the inappropriate choice of antihypertensive agents—such as non-selective β-blockers, ACE inhibitors, or the combination of ACE inhibitors with sartans (McCarthy et al., 2025)—for which clinical pharmacists recommended therapy modifications in line with current guidelines (McCarthy et al., 2025). In the treatment of arterial hypertension without other cardiovascular comorbidities, β-blockers are not considered first-line therapy; therefore, more appropriate medications, such as ACE inhibitors or sartans, were recommended (McCarthy et al., 2025). In many cases, therapies did not align with the patient’s renal or hepatic function, prompting pharmacists to recommend dose reductions or switch to safer alternatives. Unnecessary or inappropriate use of diuretics—such as regular administration of furosemide or torasemide in patients without edema—and potentially harmful drug interactions were additional issues where clinical pharmacists made recommendations (McDonagh et al., 2021; McCarthy et al., 2025). These interventions also contributed to a reduction in polypharmacy. In some cases, pharmacists recommended discontinuation of medications when hypotension was present. Hypotension is often associated with psychotropic drugs due to alpha-1 and alpha-2 receptor blockade, highlighting the need for careful medication review (Stuhec et al., 2024). As a result of these pharmacist interventions, adherence to hypertension treatment guidelines improved significantly, from 13.2% before the intervention to 72.6% after.

Adherence to treatment guidelines was initially only 3.3% in patients with diabetes mellitus, but it improved to 50.0% following pharmacist involvement. Key issues included hypoglycaemia due to excessive insulin or sulfonylureas dosing and persistent hyperglycaemia despite ongoing therapy. Most recommendations involved increasing or reducing drug doses and redistributing doses throughout the day. Other interventions primarily addressed therapy adjustments due to renal impairment, in which metformin and certain sulfonylureas are either contraindicated or require dose reduction, which aligns with the treatment guidelines (Davies et al., 2022). In some cases, more effective alternatives to acarbose—such as metformin or insulin—were recommended.

Similar results were noted in daily interdisciplinary ward rounds at a psychiatric hospital, where clinical pharmacists’ interventions improved adherence to treatment guidelines for arterial hypertension, heart failure, pain, and diabetes (Stuhec et al., 2024). Effective management of somatic comorbidities is vital for the overall management of mental disorders. Positive outcomes were further observed in two clozapine clinics, where clinical pharmacists’ recommendations enhanced the management of comorbidities such as weight, glucose levels, and lipid profiles in patients with schizophrenia treated with clozapine. Results indicate that regular medication reviews by clinical pharmacists improved the physical health monitoring of patients receiving clozapine (Spann et al., 2022).

In addition, we identified one study conducted in Greece that investigated the prevalence of inappropriate medication use for somatic comorbidities in a psychiatric clinic (Klimentidis, 2024). This pre-post interventional study included only 58 patients and reported that 75.86% were being inappropriately treated at baseline, compared to 15.52% post-intervention. The pharmacist proposed 107 interventions, of which 104 (97.2%) were accepted by physicians. However, this study did not involve a clinical pharmacist as an integrated team member, as was the case in our study. In fact, the hospital did not have a clinical pharmacist present on the ward. Furthermore, the study did not apply an advanced medication review (PCNE Type 3), which is not standardized in Greece (Urbańczyk et al., 2023). In this context, our study is the first to apply an advanced medication review (PCNE Type 3) in a psychiatric hospital setting specifically addressing somatic comorbidities.

This study has many practical implications, including clinical aspects. This study underscores the importance of medication reviews by clinical pharmacists in psychiatric hospitals for reducing medication-related problems and improving guideline adherence, particularly in polymorbid cases often excluded from randomized controlled trials. These findings support the development of new reimbursed programs and pharmaceutical services in psychiatric hospitals, building on Slovenia’s recognition as a leader in Central Europe (Stuhec et al., 2023; Urbańczyk et al., 2023).

On the other hand, this study also has many significant limitations, which are predominantly associated with the type of study (e.g., randomisation and selection bias). The study was not randomised and prospective. This would be necessary for a better level of evidence, although it would be hard in a real clinical setting with this polymorbid population. We also did not measure clinical outcomes as long-term outcomes (e.g., quality of life, survival, hospitalisations, deaths), which was not done because of the study design (retrospective study including 100 patients). This design was selected because we aimed to answer the research questions in real clinical conditions (no-RCT study), and most patients included in the medication review were polymorbid elderly individuals, who are typically excluded from RCTs. The study did not investigate the diagnoses with mental disorders, which could be a point of interest for future studies. The study also included only one psychiatric hospital, and the study could be replicated with more hospitals in Slovenia and abroad. In this study, only medication reviews were evaluated, which may underestimate the full benefits of collaboration with clinical pharmacists in a psychiatric hospital. This study also has a selection bias, as only patients with somatic comorbidities were included. However, this limitation is minor because nearly all patients reviewed had somatic comorbidities. During the study period, pharmacists conducted 106 medication reviews, of which 100 (94%) were included; only six patients did not have any of the specified somatic comorbidities. This selection also allowed us to focus our research on somatic comorbidities, which are often poorly monitored and treated, while still maintaining the calculated sample size (97). In this context, we investigated a potential approach that could be applied in other countries to optimise the management of comorbidities in inpatients with mental disorders and somatic conditions. Ethical approval for this study was obtained, and further analyses can be conducted within the framework of a new study following additional ethical approval. Future studies should explore the broader impact of clinical pharmacists as integrated team members. The results of this retrospective study, which has an uncontrolled design and potential selection bias, do not allow for a causal relationship. Therefore, these research questions should be replicated in an RCT involving multiple hospitals.

To the best of our knowledge, this is the first study to investigate the role of clinical pharmacists in conducting advanced medication reviews (PCNE Type 3) for somatic comorbidities within a psychiatric hospital setting, where clinical pharmacists are already integrated as full members of the healthcare team. The findings demonstrate that interventions by clinical pharmacists, delivered through medication reviews, reduced the number of medications, PIMs, and DDIs, while also enhancing adherence to treatment guidelines. These results underscore the important role of clinical pharmacists in optimizing therapy for these patients, despite certain limitations, such as the non-randomized design, which could be addressed in future research.

## Data Availability

The original contributions presented in the study are included in the article/Supplementary Material, further inquiries can be directed to the corresponding author.
