# Supplementary material for: Clinical pharmacist interventions in medication review for medication optimization in older hospitalized adults with mental disorders and somatic comorbidities: evidence from retrospective study
Source: Front Pharmacol. 2025 Sep 1;16:1667584. doi: 10.3389/fphar.2025.1667584 (PMC12433881; doi:10.3389/fphar.2025.1667584)
Supplement: Supplementary file 1 [file DataSheet1.docx]

**Supplementary File: Identification of Potentially Inappropriate Medications (PIMs)**

**PRISCUS List Assessment**

- The applied reference was the Potentially Inappropriate Medications in the Elderly: PRISCUS List 2.0.
- All substances listed in Table 2 of the PRISCUS document were considered.
- For medications classified as PIMs only in cases of long-term use, we evaluated them accordingly. If the duration of use before the medication review was unknown, we conservatively assumed chronic use and classified these medications as PIMs.
- Medications that were newly prescribed after the medication review and require prolonged use to meet the PIM criteria were not classified as PIMs at that point.
- For medications listed as PIMs only above a specific dosage threshold, we assessed each patient's dosage and classified them as PIMs only if the prescribed dose exceeded the stated limit. Medications prescribed below the threshold were not considered PIMs.
- In combination products, each active ingredient was evaluated individually to determine its classification according to the PRISCUS List criteria.
- Where PIM classification depended on the treatment indication, we reviewed the clinical documentation to ensure accurate classification based on the patient's medical context.

**Beers Criteria Assessment**

- The applied reference was the American Geriatrics Society (AGS) 2023 Updated Beers Criteria® for Potentially Inappropriate Medication Use in Older Adults.
- All substances listed in Table 2 of the Beers Criteria were considered for classification as potentially inappropriate medications (PIMs).
- Tables 3, 4, 5, and 6 of the Beers Criteria were not included in this analysis.
- For combination products, each active ingredient was assessed individually to determine whether it met the criteria for classification as a PIM.
- In cases where PIM classification depended on the treatment indication, we reviewed the available clinical documentation to ensure accurate classification based on the patient's medical condition.
